# Effects of the COVID-19 Health Crisis on Sports Practice, Life Quality, and Emotional Status in Spanish High-Performance Athletes

**DOI:** 10.3389/fpsyg.2021.736499

**Published:** 2021-09-27

**Authors:** Elena Conde, Luis Manuel Martínez-Aranda, Gema Sanz, Cristina López de Subijana, Antonio Sánchez-Pato, Álvaro Díaz-Aroca, Alejandro Leiva-Arcas, Juan Alfonso García-Roca, Yago Ramis, Miquel Torregrossa

**Affiliations:** ^1^Faculty of Sport, San Antonio Catholic University (UCAM), Murcia, Spain; ^2^Human Movement Neuroscience Research Group (Neuromove), (UCAM), Murcia, Spain; ^3^Department of Microbiology, Tumor and Cell Biology, Karolinska Institutet, Stockholm, Sweden; ^4^Instituto Nacional de Educación Física, Universidad Politécnica de Madrid, Madrid, Spain; ^5^Faculty of Sport, Olympic Studies Center, Catholic University of Murcia, Murcia, Spain; ^6^Departament de Psicología Bàsica, Universitat Autònoma de Barcelona, Barcelona, Spain

**Keywords:** COVID-19, elite athletes, lockdown, post-lockdown, sports performance, emotional state

## Abstract

Spain is one of the many countries highly affected by the COVID-19 crisis, establishing very restrictive measures with a complete lockdown for more than 3 months. This situation forced the complete closure of sport practice and national or international competitions, leading to a negative impact on physical and psychological health of high-performance athletes. Therefore, the objectives of this study were (a) to determine the effects of the COVID-19 health crisis on Spanish high-performance athletes in terms of sports practice, life quality, and emotional state and (b) to identify the profile with the greatest difficulties during and after the lockdown. A sample of 130 high-performance athletes aged between 18 and 34 years (67 women and 63 men) participated in this study (83.1% achieved a medal in National–International elite competitions; 86.9% were considered student-athletes). Measures included socio-demographic data through a 5-dimension *ad hoc* survey: physical activity and exercise using an adapted version from the *International Physical Activity Questionnaire (IPAQ)*; health status and limitations using an adapted version of *SF-12 Health Questionnaire*; Perceived stress *(Short-PSS)*; and Mood States (29-item *POMS*). All participants have shown a significant decrease pre–post-lockdown in both health and performance perception, especially in women, individual athletes, medalists, and student-athletes. Strong limitations of training, attention, and motivation as well as a moderate negative emotional state during lockdown were reported, in women, individual athletes, medalists, and student-athletes. Even with an improved emotional state and energy level in the post-lockdown period, moderate-to-high stress scores were reported by women and medalists. Our findings highlight the importance of paying attention to the physical and psychological health of elite athletes on three profiles: team athletes (due to social distance), student-athletes (dual-career issues), and women athletes (prevalence of implicit gender inequalities in sport).

## Introduction

In early 2020, a new viral outbreak began to spread internationally. On January 30, 2020, according to the International Health Regulations (World Health Organization from now on World Health Organization, 2020a), the outbreak was declared by World Health Organization (2020b) as “a public health emergency of international concern present in 18 countries, mainly in Europe.” Countries implemented control measures to minimize the risk of transmission and spread of the virus, such as closing schools, canceling the large meeting, suspending social and sporting events, and confining citizens to their homes. The effects of the epidemic caused by COVID-19 have been emerging as the situation evolved rapidly (Epidemiology Working Group for NCIP Epidemic Response, Chinese Center for Disease Control and Prevention, 2020).

In Spain, on 13 March, 2 days after the WHO declared COVID-19 a pandemic, the Council of Ministers decreed a “state of alarm” for the entire national territory, provided by the article 1,165 of the Constitution (Constitución española, 1978). In this process, people were confined in their homes, away from the social environment for 99 days to limit the spread of the virus, as a crucial public health measure. The effects of such lockdown raised significant mental health risks, due to an uncertainty associated with increased negative feelings (Lades et al., 2020), as well as high emotional impact (Lima et al., 2020; Xiang et al., 2020). Previous studies in Spain found that between 15.8 and 21.6% of the general population showed depressive, anxious, and/or post-traumatic symptoms during the first month of lockdown (González-Sanguino et al., 2020). From the physical health point of view (Chen et al., 2020), this situation leads to the appearance of negative effects associated with inactivity, sedentary lifestyles, increased consumption of high-calorie foods, and poor sleep quality, thereby favoring the physical deconditioning of the general population (Mera et al., 2020). From June 21, 2020, Spain ended the state of alarm and began to live in the stage known as “new normality” or post-lockdown, a battery of measures to prevent, contain, and coordinate actions to deal with the health crisis caused by COVID-19 (Royal Decree-Law 21/2020 of 9 June. BOE, 2020).

The effects of lockdown in the new situation could be specific to elite athletes compared to the general population. Athletes who have reached a high level of professionalism (Brackenridge, 2004) are committed to sport up to 40 h per week, so that sport practice has a fundamental role in their lives. Due to the global public health circumstances caused by COVID-19, the Tokyo Olympic Games scheduled for July 2020, as well as all international competitions, had to be postponed [(Schinke et al., 2020) and as (Evans et al., 2020), p. 90] noted, “*There is little doubt that the everyday lives and practices of sports participants, not least athletes, have had to change, pause or even cease as a result of the pandemic.”* The emergency health measures adopted in Spain caused an important disruption in their sporting careers, training plans, and competitions in a sudden and unforeseen manner. Both the situation of lockdown experienced and the current situation, where the athletes had to adapt very quickly, could have caused significant changes in their sporting practice, quality of life, and emotional state. This experience could have a direct impact on their sporting performance and other dimensions related to their well-being, which is even more relevant given that the Olympic Games are coming up in July 2021 and international competitions are returning.

In an athletic career, it is necessary to be aware of the transitions faced by the athlete (Stambulova and Wylleman, 2015). Special attention should be paid to the so-called non-normative transitions, due to the high level of uncertainty involved, such as the situation of the COVID-19 pandemic, and its consequences not expected. The effects that an involuntary break can have on the professional life of athletes are similar to the effects of an injury (decreasing the health status, interruption of the athletic career, loss of professional status and economic income, and so on). It is a sport-inherent fact, whether it is experienced as a catastrophe, as a challenge, or sometimes as an avoiding mechanism from stressful situations or elevated competitive anxiety (De la Vega, 2003; Stambulova et al., 2020). Therefore, the health crisis situation experienced by COVID-19 represents a challenging period for athletes, with possible effects on their lives and professional career, potentially leading to changes in several dimensions, such as physical and physiological, motor skills, psychosocial and self-identity, relationships, performance, motivation and aspirations, organizational-occupational and micro- and macro-cultural issues (Samuel et al., 2020). In this line, the postponement of the Tokyo Olympic Games has meant an important change and adjustment of their planning for some athletes, being a good opportunity to further improve their performance or recover from injuries they might have suffered (Stambulova et al., 2020; Taku and Arai, 2020). Therefore, it is necessary to understand how much the sporting practice of elite athletes has changed and the impact this might have on both their physical and psychological well-being as a consequence of a prolonged period of lockdown.

Scientific literature regarding elite athletes and their mental health during the period of social isolation (Di Fronso et al., 2020; Pons et al., 2020; Schinke et al., 2020; Stambulova et al., 2020) showed a high concern for athletes who planned not only their training for the Olympic Games, but also for other qualifying competitions. Researchers have questioned how uncertainty about missing their Olympic and other international competitions may affect the mood of athletes (Samuel et al., 2020; Schinke et al., 2020). It is argued that these changes increased the feeling of uncertainty, confusion, and frustration, and made it difficult to set concrete goals (Taku and Arai, 2020). In countries like Italy, elite athletes (Di Fronso et al., 2020) showed that the pandemic had a strong negative impact on both perceived stress and psycho-biosocial states. These harmful effects observed in both women and men were caused by the unique characteristics and demands of the emergency period. In Spain, young athletes, mainly female athletes, suffered a negative impact on their mental health during the COVID-19 lockdown (Pons et al., 2020). Also in Spain, a case study conducted with a confined Paralympic athlete in an ultra-marathon event did not show high levels of anxiety, revealing how dependent it is on the context or reality where the athlete is involved (Belinchón de Miguel et al., 2019).

Apparently, the loss of routines in lives of athletes has affected both their mental and physical states (Taku and Arai, 2020). In addition, in order to stablish and maintain an optimal performance, preparatory, control, and official competitions are an important tool (Mujika et al., 2018). Consequently, the absence of competitions has a negative impact on the athletic and competitive performance of athletes (Jukic et al., 2020). Although most athletes continued their daily training, two out of three athletes trained individually or without professional coaching. More than half of the athletes were training at a moderate exercise intensity, with a lower-than-normal training load, resulting in deconditioning, being a challenge for subsequent reconditioning in post-lockdown period (Pillay et al., 2020). In addition, attending to the motor skills dimension and the detraining or partial losses in important adaptations, the training modifications could have led to reductions in speed, technical accuracy, sensorimotor coordination, and movement flow (Tran et al., 2017; Desiderio and Bortolazzo, 2020; Jukic et al., 2020).

Concerning the well-being of athletes, it is necessary to highlight studies exploring the perceived impact on athletes due to measures of social distancing and the emotional, mental, social, and physical components related to well-being that may have been affected (Brooks et al., 2020; Gupta and McCarthy, 2021; Woodford and Bussey, 2021). The contribution of Woodford and Bussey (2021) stated that the lockdown provided the athletes with a rest period, allowing them to think about their sporting participation and make the necessary changes in their lives that would protect their well-being during the lockdown and post-lockdown periods.

In this context, it is necessary to understand the impact of lockdown on the physical and mental health of athletes in the post-lockdown period. Likewise, it is important to know how these poor training conditions may have interfered with their sporting lives (Pillay et al., 2020), as well as the existence of uncertainty due to the cancelation or delay of important competitions (Samuel et al., 2020) and the social distancing from coaches and teammates (Graupensperger et al., 2020).

Most of the studies in athletes published to date have been conducted during the lockdown period (Abenza-Cano et al., 2020; Corsini et al., 2020; Jukic et al., 2020; McGuine et al., 2021; Samuel et al., 2020; Taku and Arai, 2020) and some of them, in the final period, when athletes were very close to reactivate their sporting activity (Pillay et al., 2020), but there is little scientific evidence on the consequences perceived by athletes in the post-lockdown period and the impact it may be having on their physical and mental health and quality of life, taking into account the proximity of the Tokyo 2021 Olympic Games. Attending this need in the scientific literature, the main objectives in this study were (a) to determine the effects of the COVID-19 health crisis on Spanish elite athletes related to (a.1) sports practice, (a.2) health, life quality, and performance perception, (a.3) emotional state in the current situation of post-lockdown as well as (b) to identify the profile of the athletes with the greatest difficulties in this period, to whom the necessary attention and help should be given.

## Materials and Methods

### Participants

The participants were 130 high-performance athletes (67 women and 63 men) aged between 18 and 34 years. The 85.4% of the sample were considered top or elite athletes according to the Spanish government. The 29.2% had been in the elite for 0–3 years, 46.9% for 3–10 years, and 23.8% for more than 10 years of their sporting life. The 83.1% of the participants had achieved a gold, silver, or bronze medal in their sport discipline at least once during their time competing internationally in the elite. In total, 54.6% of the athletes belonged to individual sports, while 45.4% belonged to team sports. Finally, 86.9% of the sample were considered student-athletes. All of them gave their consent for their responses to be treated anonymously, and the University’s Research Ethics Committee approved the study, which was conducted in accordance with the Declaration of Helsinki.

### Measures

#### Sociodemographic Data

An *ad hoc* survey was created in order to collect relevant data according to the objectives of this study. This survey was composed by items related to (a) gender, (b) best sporting result obtained (medalists, non-medalists), (c) sporting discipline practiced (individual-team sports), (d) student-athlete, and (e) high-performance sporting expertise.

#### Physical Activity and Exercise

All information related to the amount of physical activity and resting time performed by the participants was collected. For this purpose, the short version proposed by the Junta de Andalucía of the *International Physical Activity Questionnaire (IPAQ*; Mantilla and Gómez-Conesa, 2007) was used. This shortened version consisted of seven items and provided information about the time respondents spent in moderate- and vigorous-intensity activities, walking, and resting. In this adapted version, several items were duplicated in order to collect data from pre-lockdown and post-lockdown periods. Additional items were included for reporting the resting days and hours, since three time-point (pre–during–post-lockdown) was collected. Therefore, a total of 18 items composed this adapted questionnaire to the participants. The Cronbach’s alpha coefficient reported acceptable levels of reliability, with values ∼0.80. This instrument allowed monitoring the physical activity prevalence of the adult population aged 18–65 years.

#### Health Status and Limitations Questionnaire

An adapted questionnaire based on the original *SF-12 Health* Questionnaire (Ware et al., 1996; Gandek et al., 1998) translated into Spanish by Alonso et al. (1998) and Vilagut et al. (2005) was used. This instrument consisted of 12 items from the eight dimensions of the original *SF-36* (Ware and Sherbourne, 1992). For this occasion, an adapted questionnaire was expanded and applied in order to collect pre–post data (dimensions 1 and 2), during and post-lockdown periods (dimensions 39), consisting in 20 items related to nine dimensions. The number of response options assessing intensity or frequency ranged from three to six, depending on the dimension: *(1) health perception, (2) performance perception (ranging both between 0 = bad and 4 = excellent), (3) degree of limitation in training (from 0 = No, I trained what I really wanted to 4 = Yes, I trained a way less than I wanted to), (4-6) Limitations of training, attention and motivation due to negative emotional causes (Yes, No, Maybe), and finally, (7-8) Positive-negative emotional state and (9) energy levels (from 0 = never to 5 = always).* Cronbach’s alpha coefficient reported values of reliability between 0.78 and 0.96. This instrument provided a profile of the health status of participants and is one of the most widely used generic scales in both descriptive and evaluative studies.

#### Perceived Stress

A shortened version of the Spanish version of the *Perceived Stress Scale* (PSS; Cohen et al., 1983) designed by Remor and Carrobles (2001) was used. This scale is a self-report instrument that assesses the level of stress perceived during the last month. The shortened version used in this study has 12 items with a five-point scale response format (0 = never, 1 = hardly ever, 2 = occasionally, 3 = often, 4 = very often). The total *PSS* score is obtained by inverting the scores of items 4, 5, 6, 7, 9, and 11 (as follows: 0 = 4, 1 = 3, 2 = 2, 3 = 1, and 4 = 0) and then adding the 12 items together. The direct score obtained indicates that a higher score corresponds to a higher level of perceived stress. Cronbach’s alpha coefficient reported the values of reliability between 0.78 and 0.82. The PSS was completed concerning the post-lockdown period.

#### Mood States

The *Profile of Mood States (POMS*) in its original reduced version (McNair et al., 1992), composed of 29 items and validated in Spanish by Fuentes et al. (1995), was used. This version includes a Likert-type scale with values ranging from 0 (not at all) to 4 (very much) to assess the factors Fatigue, Depression, Tension, Hostility, and Vigor. The Cronbach’s alpha showed values between 0.70 and 0.83. This instrument is useful for assessing mood and its relationship to sport (Abenza et al., 2010). Participants completed the POMS questionnaire regarding the post-lockdown period.

#### Procedure

After obtaining the approval of the Ethics Committee of the University, the online tool was designed through the *Google Forms* platform using all the aforementioned questionnaires. The online survey was sent by email following a convenience sampling strategy, to 2,136 athletes from different sports disciplines being registered in the COE (Spanish Olympic Committee). In total, 130 elite athletes (83.1% won international medals) completed the whole online survey agreeing to participate in the present study. Through human resources contact, coaches from multiple sports disciplines were encouraged to discuss with their athletes the importance of conducting this type of study and to raise awareness of the difficulties that elite athletes have experienced both physically and psychologically, during this unusual and uncertain period. During 5 weeks, the project team was in contact with the supervisor for the athlete recruitment in order to receive information about the process. Responses to the online questionnaire by the athletes were provided in a single session and without any interruption until all the tests were completed. The completion of the online survey was performed in a controlled manner and in the presence of the respective coaches. The athletes answered the IPAQ questionnaire according to pre- and post-lockdown periods, except for resting days and hours with three time-point questions: (a) PRE (before the lockdown); (b) DURING (during the lockdown period); and (c) POST (after the lockdown). For the questionnaire related to health status and limitations, only two points (pre vs. post or during vs. post) were taken into consideration. The remaining questionnaires (perceived stress and mood states) were only related to the post-lockdown period. Athletes who agreed to participate were informed of the confidential and voluntary nature of the study as well as the absence of any reward for participation. When answering the questionnaire, all of them had accumulated 99 days of lockdown. Finally, in appreciation of their collaboration, a final report was sent with the main findings of the study.

#### Data Analysis

Descriptive statistics were performed on all participants in the study. A Lilliefors test of normality was applied (>50 samples), yielding non-normal results for all variables except Stress score. Median and interquartile ranges were used for non-normal continuous variables, and mean (± standard deviation) was used for Stress score variable. The N and percentages were used for descriptive variables as well. Comparisons for each variable type were assessed based on the following categorical variables: gender, best result achieved, modality, student-athlete, and high-performance expertise. Wilcoxon’s rank-sum test was used for continuous variables if non-normally distributed; otherwise, a *t*-test was applied. Additionally, paired Wilcoxon signed-rank test was used to assess changes within pre vs. during or during vs. post and pre vs. post COVID-19 lockdown when available. Chi-square test was used for categorical variables. Wilcoxon’s effect size (ES) was calculated for non-normal variables; otherwise, Cohen’s D was used. Differences at *p <* 0.05 level were considered statistically significant. All statistical analyses and plots were performed using the packages nortest (Gross and Ligges, 2015) and rstatix (R version 4.0.3) (Kassambara, 2021). Correlation analyses were performed using the R “rquery. cormat”^1^ function and corrplot package (Wei and Simko, 2017).

## Results

The descriptive data from all the variables are shown in Supplementary Table 1. These results are presented as N and percentages, median and interquartile range, or mean and standard deviation, depending on the type of variable. Five comparative categorical groups were established for analyzing all the results in this study: gender, modality, best result achieved, student-athlete, and high-performance expertise.

### Days and Hours Pre-(during)–Post-lockdown: Training, Moderate Physical Activity, Walking, and Resting

A paired Wilcoxon signed-rank test revealed significant differences in days of resting in pre vs. during and during vs. post for all of the groups analyzed (*p* < 0.001). Attending to the categorical variables, men increased from 2 to 6 days of rest during the lockdown, and then returned at 2–3 days in the post-lockdown period. Team sports use more resting days in each pre–during–post stage compared to individual sports (*p < 0*.01). Student-athletes increased resting days during lockdown to a lower extent compared to non-athletes, regained their pre-lockdown habits later on (*p* < 0.01). From a general perspective, sportsmen and women belonging to team sports and with lower competitive level retained 1 day more for resting in the post-lockdown period compared to pre-lockdown.

Concerning hours of training and moderate physical activity, changes were observed in medalist athletes (podium) (*p* = 0.008), in individual sports (*p* = 0.003), and in athletes with medium-broad expertise in elite (*p* = 0.032), significantly decreasing their daily and weekly training hours between pre- and post-lockdown.

Significant differences for hours of resting pre vs. during, during vs. post and pre vs. post, were reported for the whole sample and by subcategories, especially for both genders, medalist athletes, both individual and team sports, student-athletes, and athletes with 3–10 years of experience in the elite (*p* < 0.001). The aforementioned samples considerably increased the hours of rest per day during lockdown, where the intervals of 3–5 h and +5 h per day were greatly increased. In the post-lockdown period, the resting hours were generally somewhat higher than those shown in the pre-lockdown period.

### Health and Performance Perception

**FIGURE 1:**
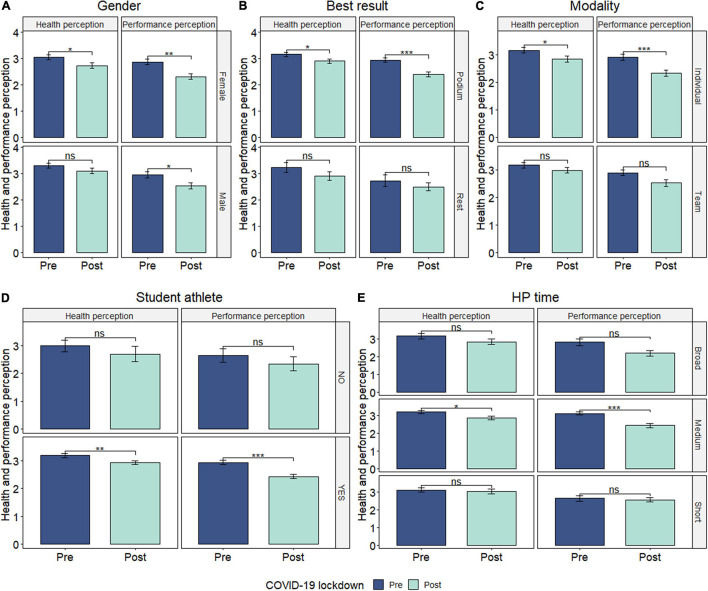
**(A–E)** Health and performance perception by gender, best result achieved, modality, and student-athlete condition. Data presented as mean ± SD. *Significant at level 0.05; **significant at level 0.01; ***significant at level 0.001.

Regarding the health perception in the post-lockdown period, significant differences between genders were revealed, where women’s score was significantly lower than men (*p* = 0.02, small ES). Both genders, male (*p* = 0.02, small ES), and female (*p* = 0.001, moderate ES), showed a significant decrease in the performance perception pre–post-lockdown (Figure 1A).

Athletes competing in individual sports, the group of medalists, student-athletes, and those with 3–10 years of expertise have shown a significant decrease in both health perception (*p* = 0.02, small ES; *p* = 0.01, small ES; *p* = 0.003, small ES; *p* = 0.02, small ES, respectively) and performance perception (*p* < 0.001, moderate ES; *p* < 0.001, small ES; *p* < 0.001, small ES; *p* < 0.001, moderate ES, respectively) pre–post-lockdown (Figures 1B–E).

### Degree of Limitation in Training During–Post-lockdown

The degree of limitation in training was compared during and post-lockdown for all the categorical variables. A paired Wilcoxon signed-rank test indicated that these differences were significant (*p* < 0.001, large ES).

### Limitations of Training, Attention, and Motivation Due to Negative Emotional Causes

A chi-squared test found that there was a statistically significant association between the limitations of training [*χ*^2^_(__2)_ = 36.847, *p* < 0.001], attention [*χ*^2^_(__2)_ = 25.469, *p* < 0.001] as well as motivation [*χ*^2^_(__2)_ = 50.730, *p* < 0.001] due to negative emotional causes, and the lockdown status. A higher number of participants reported limitations during lockdown period but not after.

### Positive/Negative Emotional State and Energy Levels During–Post-lockdown

**FIGURE 2:**
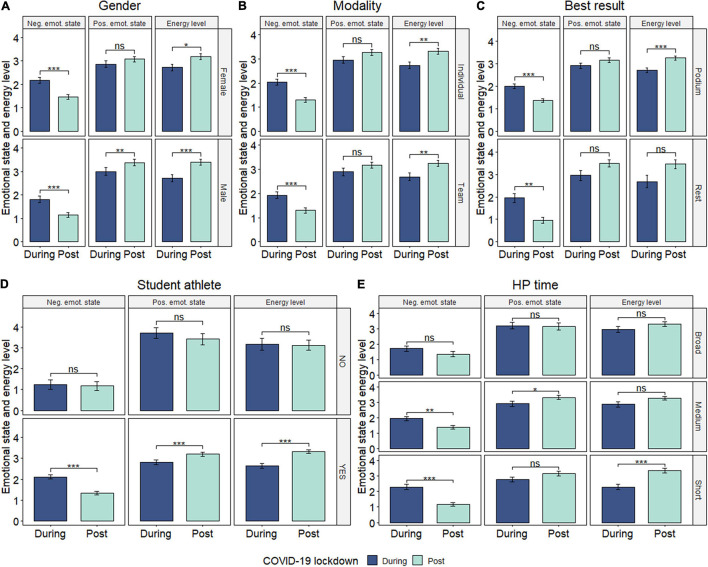
**(A–E)** Emotional and energy state by gender, modality, student-athlete condition, and high-performance expertise. Data presented as mean ± SD. *Significant at level 0.05; **significant at level 0.01; ***significant at level 0.001.

Concerning the negative emotional state, Figures 2A–E show intermediate levels during lockdown, dropping significantly to lower levels in the post-lockdown period for both genders (female–male, *p* < 0.001, moderate ES, being more pronounced in men); both modalities (individual-team; *p* < 0.001, moderate ES and *p* < 0.001, small ES, respectively); best result (podium-rest; *p* < 0.001, small ES and *p* < 0.01, large ES, respectively); student-athletes (*p* < 0.001, moderate ES, significantly higher during lockdown compared to non-student-athletes), and short and medium high performance (HP) sporting expertise (*p* < 0.001, large ES and *p* < 0.01, small ES, respectively).

The positive emotional state was moderate to high, increasing in the post-lockdown compared to lockdown for only male (*p* < 0.01, small ES); student-athletes (*p* < 0.001, small ES, significantly lower during lockdown compared to non-student-athletes); and athletes with (medium) 3–10 years of experience in the elite (*p* < 0.05, small ES) (Figures 2A–E).

The participants in this study also showed moderate energy levels during lockdown, increasing significantly to higher levels in the post-lockdown for both genders (*p* < 0.05 and *p* < 0.001, small ES, for female and male, respectively); individual and team athletes (*p* < 0.01, small ES); medalists (*p* < 0.001, small ES); student-athletes (*p* < 0.001, moderate ES); and short sporting experience (*p* < 0.001, large ES) (Figures 2A–E).

### Stress and Mood States

**FIGURE 3:**
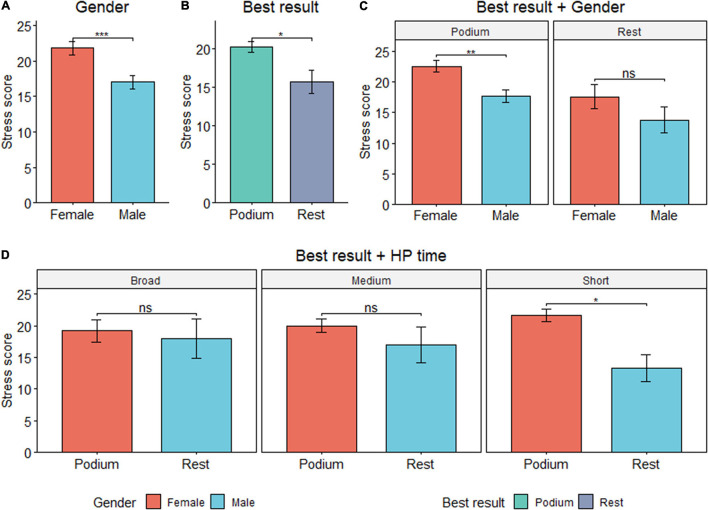
**(A–D)** Stress scores by gender, best result achieved, and high-performance expertise. Data presented as mean ± S D. *Significant at level 0.05; **significant at level 0.01; ***significant at level 0.001.

A *t*-test revealed significant differences between genders for stress scores (*p* = 0.001, moderate ES) and in medalists, with higher scores compared to non-medalists (*p* = 0.01, moderate ES). When combining both variables, women still show significantly higher levels, but only within those competitors with the best result (podium) (*p* = 0.001, moderate ES). In addition, women showed higher scores compared to men in team sports (*p* = 0.008), student-athletes (*p* = 0.004), and medium sporting expertise (3–10 years) (*p* = 0.002) (Figures 3A–C).

By combining best result and HP sporting expertise, the medalists with 0–3 years of expertise showed higher stress scores compared to non-medalists (*p* = 0.01, large ES). In general terms, as sporting expertise increases, the differences between medalists and non-medalists equalize and become non-significant after 3–10 years of experience in the elite (Figure 3D).

**FIGURE 4:**
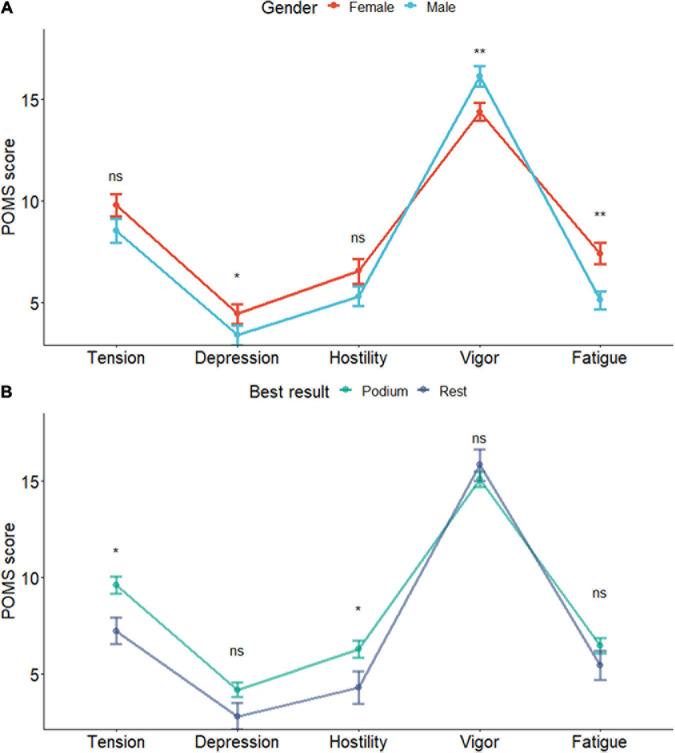
**(A,B)** Profile of mood states by gender and best result achieved. Data presented as mean ± SD. *Significant at level 0.05; **significant at level 0.01.

Concerning the mood states by gender, a Wilcoxon signed-rank test revealed significant differences in depression (*p* = 0.04) and fatigue (*p* = 0.003) with higher scores for women, and vigor with higher scores for men (*p* = 0.008). Those with better results have a slightly more negative profile, showing higher levels of tension (*p* = 0.02) and hostility (*p* = 0.04) than non-medalists. They generally score a little higher on the negative dimensions of the profile and lower on vigor compared to non-medalists. No significant differences were found for any factor regarding other categorical variables (Figures 4A,B).

### Correlational Study

**FIGURE 5:**
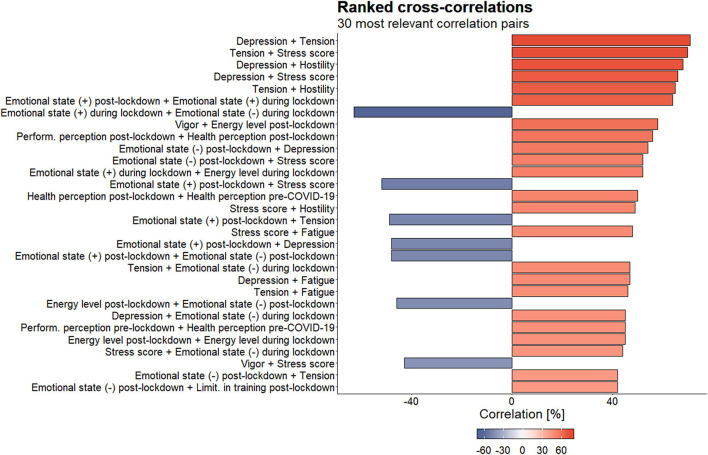
Top 30 ranked cross-correlations for the stress, POMS, emotional state and energy level, health and performance perception, and limitation in training variables. Red and blue colors correspond to positive and negative correlations, respectively. Size of the bar and color intensity are proportional to the correlation coefficients.

The 30 most relevant ranked cross-correlations are shown in Figure 5 and Supplementary Figure 1. Mood states, positive and negative emotional states, energy levels, stress scores, health-performance perception, and degree of limitation in training were the variables yielding the most significant negative–positive correlations for all participants.

## Discussion

The health crisis caused by COVID-19 raised a series of hitherto unknown circumstances in the world of elite sport, as the population had to be confined to their homes (for more than 3 months, in the case of Spain). It also meant the delay and/or cancelation of sporting events all over the world, with the postponement of the Olympic Games for the first time in its history, being an example of the magnitude of this crisis worldwide. A large number of retrospective studies were carried out during the lockdown period, trying to understand the problems athletes were facing and how they could affect their physical and mental health. To complement these studies and the problems that have become evident, it is necessary to know what consequences have been produced on the lives months of athletes after exceptional circumstances. For this purpose, the present results extend previous knowledge on the consequences of the COVID-19 pandemic by providing new evidence on the physical status, quality of life, and mental health of Spanish top-level athletes who, after a long lockdown period, had to return to their sporting lives in a new context.

Attending to physical health, the major increase in the resting time that Spanish athletes dedicated during the lockdown period seems to be a relevant aspect to be taken into account. According to previous studies with elite athletes (Pillay et al., 2020), a significant increase in rest time during lockdown characterized by sedentary activities is claimed. Although athletes were assigned to individual training plans during the lockdown, they reduced and adapted those training in order to accommodate self-isolation and due to the closure of sports facilities and gyms (Mohr et al., 2020). In this case, both genders significantly increased their rest days in the lockdown period compared to the previous period. In post-lockdown period, it is observed that athletes, due to the progressive incorporation to their daily activities, even with a decrease in rest days compared to those in lockdown, continued to rest around 1 day more than pre-lockdown period, indicating that certain habits are not recovered quickly or possibly that the return to training has been carried out in a progressive and scheduled manner by the trainers, aware that after a quarantine, athletes are more likely to get injured (Jukic et al., 2020). In this line, studies focused on young athletes in the United States reported that women were less physically active than men during the lockdown period and had more sedentary behaviors, which could be related to factors such as lack of social interaction, increased economic uncertainty (McGuine et al., 2021), as well as the existence of implicit gender inequalities during the pandemic (Evans et al., 2020).

It should also be noted the rest days of team sports athletes, which were longer compared to individual sports athletes during the lockdown period, coinciding with the study by Pons et al. (2020) focused on young athletes in Spain. Lower levels of physical activity were observed in favor of sedentary activities, due to greater limitations for practicing, as well as the possibility that individual sports athletes could continue to participate in their sports when physical distance restrictions were established and, therefore, were not affected to the same extent as team sports athletes.

Also, noteworthy are the results in student-athletes, characterized by pursuing academic studies at the same time as their sporting careers and are framed within the so-called dual career (Pallarés et al., 2011), where a high level of effort and many hours of dedication are required to achieve such a conciliation. Student-athletes maintain a complex relationship between studies and sport, maintaining a special organization to achieve academic and sporting goals simultaneously (Álvarez Pérez and López Aguilar, 2012; López de Subijana et al., 2015; Blanco García and Burillo Naranjo, 2017). During the lockdown, they also spent more time on rest-related activities (days and hours) compared to the pre-lockdown normality. Similar studies (Meza and López, 2020) also stated that student-athletes during lockdown significantly decreased their physical activity (33.6%) in favor of more sedentary activities compared to the pre-lockdown physical activity.

In any case, they duplicated the practice of physical activity compared to non-athlete university students. Apparently, despite the loss of physical activity, the student-athletes followed the recommendations to exercise at home during the lockdown, but similar to the results of the present research, the sedentary activities could be related to increasing the time dedicated to studies, with the aim of synchronizing the learning processes in their future profession.

On the contrary, these statements would disagree with the results reported by Izzicupo et al. (2021), a study carried out during lockdown in several European countries focused on student-athletes. An overall reduction in study time was observed during the lockdown, even though student-athletes living in countries under severe contagion (as is the case in Spain) were more involved in studies than those living in countries experiencing mild contagion, a reduction that the authors found contradictory when considering the greater availability of time at home.

Related to progressively returning to sport, it is observed that there are differences between the hours that athletes dedicated to training before the lockdown and in the post-lockdown. The athletes with the highest competitive level, in individual sports and with medium and broad experience in the elite, significantly reduced their daily and weekly training hours after the lockdown by returning to training. For this reason, the main responsible for the athletes’ return to training and competitions should have specific knowledge of the different profiles and how the COVID-19 health emergency situation may have influenced their lives. Along the same lines, Desiderio and Bortolazzo (2020) stated that *“activity must be resumed slowly and by listening to the athlete, as there is still uncertainty as to whether we will return to the old habits of the old ‘normality,’ or whether the pandemic will give rise to a new stage with new lifestyles that will define the future”* (p. 54).

The health perception of athletes in the post-lockdown period after the difficulties experienced during the lockdown period, is a factor that has been taken into account through two summary components: physical and psychological (Vilagut et al., 2008). Accordingly, differences were found between genders, with women scoring significantly lower than men, compared to their perception in pre-lockdown. Related to the performance perception, both genders, but more pronounced in females, showed a significant decrease comparing pre–post lockdown periods.

Although the health and performance perception in athletes after the lockdown period has not yet been extensively studied, a study carried out during lockdown with more than 13,000 youth athletes (McGuine et al., 2021), states that female athletes described lower life quality levels than males. In addition, following the study by Bowes et al. (2020), it seems that the gender should be considered in those periods, as women perceived to have less access to sports equipment than men and were also more affected by reduced or even suspended salaries, affecting their life quality and performance perception. According to Baldwin (2020), the pandemic has had a disproportionate impact on elite sport of women and has increased inequality, with possible spillover effects for the future. Therefore, it seems necessary to increase the number of studies focused on the problems experienced by female athletes during lockdown and the post-lockdown period.

Considering the competitive level of athletes, those with higher level (medalists) reported a significant decrease in the health and performance perception compared to pre-lockdown. It could be interpreted that these athletes were those who had Olympic qualification as one of their next objectives. Therefore, after 4 years of preparation, the postponement was an added stressor. In this line, studies affirm that this situation could cause a loss of concentration, motivation, and the desire to continue preparing for the Olympics with the same energy as before, affecting, among other variables, their health and performance perception (Clemente-Suárez et al., 2020). It should be noted that individual-sports athletes perceived themselves to be less healthy than team sports athletes. Student-athletes also perceived a decrease in the health and performance perception, as well as athletes with an average experience (3–10 years in elite sport).

The health of athletes was possibly also influenced by emotional problems experienced during the lockdown period, the reduced time dedicated to training, and the training performance with less attention and motivation compared to pre-lockdown. Social distancing seems to be one of the main reasons for the appearance of such problems (Burgos et al., 2020). Studies performed during lockdown (Graupensperger et al., 2021) affirm the relevance of the social connection and support of teammates in the emotional state of athletes and its relation to sport identity, understood as the strength that makes people identify with and accept their role as athletes (Brewer et al., 1993).

This perceived loss of social connection and support (Brewer et al., 2010) could increase the risk of depression (Sanders and Stevinson, 2017), by threatening their identity. Chilean cyclists during lockdown (Burgos et al., 2020) emphasized the anxiety caused by being unable to train on a regular basis, leading them to implement strategies to generate motivation and not lose the competitive level they already had.

Focusing on the post-lockdown period, differences between the limitations experienced in training during lockdown (high degree) and the post-lockdown (low degree) were observed for both genders, also reported by medalists, individual sports, student-athletes, and those with more experience in the elite. It seems clear then the importance of maintaining adequate levels of physical activity in professional athletes, as in addition to positive physiological effects, sports practice enhances psychological well-being. Moreover, strategies should be incorporated to reduce stress and anxiety about returning to competition, respecting each individual’s own time, avoiding injuries and frustrations to quickly reach the competitive level they showed prior to lockdown (Olmedilla et al., 2014; Kalazich Rosales et al., 2020; Mera et al., 2020). Regarding emotional state of athletes for the entire sample (both genders; medalists or non-medalists; individual-team sports; short and medium sporting experience), moderate values in the negative emotional state during the lockdown period were found, being positively correlated with the levels of tension, depression, and stress reported later on. Despite this, they maintained moderately high levels of positivity and energy, possibly due to the novelty of the situation in the first 5–6 weeks, the increased time dedicated to entertainment and the release from work responsibilities in many cases.

These negative emotional values decreased significantly in the post-lockdown period, especially in men, medalists, student-athletes, both modalities (individual-team), and athletes with 0–3 years of experience. This correlates with a decrease in training limitations, and an inverse correlation in the positive emotional state and energy level, both increasing significantly in the post-lockdown period. This is possibly linked to being able to get back to socializing, training again with the opportunity to compete in a short-term period, resuming studies and leading a regular life after months of lockdown.

Furthermore, it is important to highlight those fluctuations in stress and modifications in exercise routines, especially when drastic, could result in sports injuries or illnesses (Brink et al., 2010; Wadey et al., 2013; Ivarsson et al., 2017). According to these statements, studies on the effect on the athletes’ emotional state of unforeseen events that can slow down a sports career, such as an injury (Olmedilla et al., 2014) or an involuntary lockout (Samuel et al., 2020), affirm that injuries negatively affect the mood, causing important changes and limiting sports competence and the derived performance (Montero et al., 2014).

The so-called Coronavirus experience (Samuel et al., 2020) is understood as a longitudinal, multifaceted, unpredictable, and uncontrolled change event where athletes may experience various emotional responses (positive, negative, and/or neutral). Therefore, it seems necessary to study the emotional state of athletes and related issues, even a period after the lockdown. Likewise, other studies argue that the pandemic and associated effects must be looked at in a complex and multidisciplinary way, not focusing exclusively on a biomedical approach and the responsibility of each country’s health ministries, as only one of the dimensions affecting athletes will be addressed (Burgos et al., 2020; Stambulova et al., 2020).

As discussed above, and now related to the emotional state, student-athletes should be particularly considered since their behavior is different compared to other athletes not combining academic and sporting life. Significant differences were reported in the negative emotional state, with student-athletes showing a worse baseline level during lockdown, suggesting a worse post-lockdown emotional state, but actually the results showed a significant drop afterward, correlating with the increase in positivity and energy. These results could mean that the student athletes took advantage of the lockdown period, to spend more time than usual on their studies instead of training, whereas, after return to training, they experienced a better emotional state, as they can continue their normal academic activity as well as training and competition, feeling more competent and controlling the situation. In fact, non-student-athletes showed an inverse behavior, being affected by the situation to a greater extent, maintaining their emotional state unchanged in the post-lockdown period.

As highlighted by other studies (Samuel et al., 2020; Stambulova et al., 2020), COVID-19 lockdown can place unexpected demands on athletes who combine an academic and sporting career and, therefore, have negative consequences on their emotional state. This can be explained in our sample by the stress levels reported, probably due to the new challenge of restarting all the activity again, taking into account their dual career status.

Student-athletes have reported higher values of negative emotional state during the lockdown, similarly to Toresdahl and Asif (2020) who stated that the cancelation of training and competitions could cause pain, stress, anxiety, frustration, and sadness in athletes, where students in countries with severe contagion, as in the case of Spain, may have increased vulnerability and support needs to cope with distance education and digital skills (Izzicupo et al., 2021).

The results on stress and mood of athletes in the post-lockdown period showed significant differences between genders and best sporting results. Women showed higher levels of depression and fatigue, as well as higher levels of stress, especially those sportswomen with better results. On the other hand, men had higher values for vigor. Once again, the present study shows that women experienced more negative consequences of lockdown, possibly affecting their mental health and, therefore, their personal life and sporting performance in the period of post-lockdown. Clarkson et al. (2020) suggest that the pressure on women’s sport during the pandemic is different compared to men and that the lack of financial security, as well as feelings of threat and uncertainty, was widespread for players at all levels of women’s football. Completing these findings, Bowes et al. (2020) affirm that the negative impact felt by female athletes is not a direct result from those alongside whom they work (coaches, psychologists, and larger support networks) but from the broader sport structures, historically rooted in gender inequality.

According to Rowe (2020), the virus did not cause sporting inequalities, but has exacerbated them across gender lines. In Spain, the study conducted by Pons et al. (2020) with young student-athletes also states that the group of female athletes suffered a greater impact, especially those in higher academic courses, with a lower socioeconomic status and worse training conditions during confinement. Di Fronso et al. (2020) suggest future research lines to be explored, since they have revealed a more negative impact of COVID-19 confinement on Italian female athletes. The sporting level (medalist athletes) seems to be another indicator to be considered in the profiles of athletes with more difficulties, experiencing higher stress levels and hostility than non-medalists.

## Research Limitations

Carrying out a cross-sectional study to find out the situation of athletes at different times of the health crisis could find limitations in the results obtained, because the responses were obtained in the post-confinement period. A longitudinal study could have been presented for the most appropriate in this case, but the uncertainty in the duration of the periods lived, as well as the difficulties that the athletes were facing, were the reason for carrying out the study once the confinement had passed and efforts were being made to regain normalcy, even with a large number of limitations.

In addition, other limitations to consider would be the inclusion of the questionnaires used in the present study in a single survey, which could mean that it was too long for the athletes to answer, as well as the inability to take the data in person due to mobility limitations that characterized the post-confinement stage in Spain.

## Conclusion

This study shows how the health crisis caused by COVID-19 altered the sporting practice, life quality, and emotional state of Spanish elite athletes during the lockdown and in the subsequent post-lockdown period. Concerning the sporting practice, even with the advice of coaches during the lockdown, there were modifications due to the restrictions and the closure of sports facilities. After returning to the new situation, sporting activity did not return to normal levels immediately, since training was carried out in a progressive and scheduled manner by professionals, well aware that training had to be adapted to the circumstances. The health and performance perception were other altered factors in the athletes, especially in student-athletes and women groups. In addition, athletes with a higher competitive level suffering greater uncertainty due to the postponement of competition had an important decrease in both perceptions that should be considered. Furthermore, the health of athletes was also influenced by emotional problems, leading them to perform their training sessions with less attention and motivation compared to before the health crisis. However, the post-confinement period led to a decrease in the negative emotional state of the athletes, while at the same time increasing their positive emotional state and energy level in order to face their tasks. On the other hand, the stress level remained moderately high, due to the workload to be recovered, especially concerning the competitive and academic field for student-athletes.

We highlight the identification of profiles of different athletes to be considered, due to the consequences and difficulties caused by the health crisis on their lives. First, Spanish women athletes could be a group to be specifically approached that might indicate the prevalence of implicit gender inequalities in sport, becoming even more evident in a global crisis situation. Our findings could be related to less support received from organizations, as well as to other gender-related difficulties (less access to sports equipment, reduction or suspension of salaries, and so on) that should be further studied. Considering the physical and emotional health of female athletes, it appears to have been impaired, potentially affecting their lives and sports performance. During the last two Olympic Games, Spanish women have won more medals (52.4 and 64.7% in London and Rio, respectively). Therefore, it would be necessary to increase the research, in order to know the real impact of the pandemic on the elite sport of women in Spain, as well as the current and future consequences of the pandemic. Second, Spanish athletes who practiced team sports had more limitations when training both during and in post-lockdown periods, mainly due to the social distance, something that should clearly be considered. Third, student-athletes, who found the lockdown an opportunity to focus more time on their academic careers, leading to an increase in more sedentary activities, with possible implications for their sports performance on the one hand, but advances for their lives outside of sport as well.

## Data Availability Statement

The original contributions presented in the study are included in the article/Supplementary Material, further inquiries can be directed to the corresponding author/s.

## Ethics Statement

The studies involving human participants were reviewed and approved by the San Antonio Catholic University (UCAM), Murcia, Spain. The patients/participants provided their written informed consent to participate in this study.

## Author Contributions

EC, CLdS, and MT participated in the design of the researchproject. EC, LMMA, CLdS, and MT participated in the surveydesign and adjustments. ASP, AL, and JAGR participated in thesubject recruiting and data collection process. LMMA and GSparticipated in the data analysis and results. EC, LMMA, GS, andADA participated in the preparation of the original manuscript. EC, LMMA, GS, YR, CLdS, and MT participated in the revised version of the manuscript. All authors contributed to the article and approved the submitted version.

## Conflict of Interest

The authors declare that the research was conducted in the absence of any commercial or financial relationships that could be construed as a potential conflict of interest.

## Publisher’s Note

All claims expressed in this article are solely those of the authors and do not necessarily represent those of their affiliated organizations, or those of the publisher, the editors and the reviewers. Any product that may be evaluated in this article, or claim that may be made by its manufacturer, is not guaranteed or endorsed by the publisher.

## Footnotes

1
http://www.sthda.com/upload/rquery_cormat.r

## References

[B1] Abenza L. Olmedilla A. Ortega E. Ato M. García-Más A. (2010). Análisis de la relación entre el estado de ánimo y las conductas de adherencia en deportistas lesionados. *An. Psicol.* 26 159–168.

[B2] Abenza-Cano L. Leiva-Arcas A. Vaquero-Cristóbal R. García-Roca J. A. Meroño L. Sánchez-Pato A. (2020). Effect of coronavirus disease 2019 (COVID-19) on elite spanish student-athletes’ perception of the dual career. *Front. Psychol.* 11:620042. 10.3389/fpsyg.2020.620042 PMC7779607 33408676

[B3] Alonso J. Regidor E. Barrio G. Prieto L. Rodríguez C. de la Fuente L. (1998). Valores poblacionales de referencia de la versión española del Cuestionario de Salud SF-36. *Med. Care* 111 410–416. 9834913

[B4] Álvarez Pérez P. López Aguilar D. (2012). Armonización entre proceso de aprendizaje y práctica deportiva en universitarios deportistas de alto nivel. (Harmonization Between Learning Process and Sport Practice in High Level University Athletes). *Cultura Ciencia Deport.* 7 201–212. 10.12800/ccd.v7i21.85

[B5] Baldwin A. (2020). *Pandemic hit Women’s Sport Much More than Men’s – UK Parliamentary Report.* Available online at: https://www.reuters.com/article/us-health-coronavirus-sport-britain-idUSKCN24N34Q (accessed May 5, 2021).

[B6] Belinchón de Miguel P. Ruisoto-Palomera P. Clemente-Suárez V. J. (2019). Psychophysiological stress response of a Paralympic athlete during an ultra-endurance event. A case study. *J. Med. Syst.* 43:70. 10.1007/s10916-019-1188-6 30737600

[B7] Blanco García P. Burillo Naranjo P. (2017). Los deportistas de élite en el sistema universitario español (Elite athletes in the Spanish university system). *Retos* 0 162–168.

[B8] BOE (2020). Real Decreto-Ley 21/2020, de 9 de Junio, de Medidas Urgentes de Prevención, Contención y Coordinación Para Hacer Frente a la Crisis Sanitaria Ocasionada Por el COVID-19. Madrid: Gobierno de España

[B9] Bowes A. Lomax L. Piasecki J. (2020). The impact of the COVID-19 lockdown on elite sportswomen. *Manag. Sport Leisure* 1–17. 10.1080/23750472.2020.1825988

[B10] Brackenridge C. (2004). Women and children first? Child abuse and child protection in sport. *Sport in society* 7 322–337. 10.1080/1743043042000291668

[B11] Brewer B. W. Cornelius A. E. Stephan Y. Van Raalte J. (2010). Self-protective changes in athletic identity following anterior cruciate ligament reconstruction. *Psychol. Sport Exerc.* 11 1–5. 10.1016/j.psychsport.2009.09.005 20161402 PMC2783627

[B12] Brewer B. W. Van Raalte J. L. Linder D. E. (1993). Athletic identity: hercules’ muscles or achilles heel? *Int. J. Sport psychol.* 24 237–254.

[B13] Brink M. S. Visscher C. Arends S. Zwerver J. Post W. J. Lemmink K. A. (2010). Monitoring stress and recovery: new insights for the prevention of injuries and illnesses in elite youth soccer players. *Br. J. Sports Med.* 44 809–815. 10.1136/bjsm.2009.069476 20511621

[B14] Brooks S. K. Webster R. K. Smith L. E. Woodland L. Wessely S. Greenberg N. (2020). The psychological impact of quarantine and how to reduce it: rapid review of the evidence. *Lancet* 395 912–920. 10.1016/S0140-6736(20)30460-8 32112714 PMC7158942

[B15] Burgos A. V. Leiva G. M. López P. V. (2020). Percepción de deportistas chilenos respecto a los efectos emocionales del distanciamiento social. *Epidemiol. Acción* 28 28–34

[B16] Chen P. Mao L. Nassis G. P. Harmer P. Ainsworth B. E. Li F. (2020). Wuhan coronavirus (2019-nCoV): the need to maintain regular physical activity while taking precautions. *J. Sport Health Sci.* 9:103. 10.1016/j.jshs.2020.02.001 32099716 PMC7031771

[B17] Clarkson B. G. Culvin A. Pope S. Parry K. D. (2020). Covid-19: reflections on threat and uncertainty for the future of elite women’s football in England. *Manag. Sport Leisure* 1–12. 10.1080/23750472.2020.1766377

[B18] Clemente-Suárez V. J. Fuentes-García J. P. de la Vega Marcos R. Martínez Patiño M. J. (2020). Modulators of the personal and professional threat perception of Olympic athletes in the actual COVID-19 crisis. *Front. Psychol.* 11:1985. 10.3389/fpsyg.2020.01985 PMC7419607 32849157

[B19] Cohen S. Kamarck T. Mermelstein R. (1983). A global measure of perceived stress. *Journal of Health and Social Behavior* 24 385–396. 10.2307/2136404 6668417

[B20] Corsini A. Bisciotti G. N. Eirale C. Volpi P. (2020). Football cannot restart soon during the COVID-19 emergency! A critical perspective from the Italian experience and a call for action. *Br. J. Sports Med.* 54 1186–1187. 32209554 10.1136/bjsports-2020-102306

[B21] Constitución española (1978). *Constitución Española.* Madrid: Constitución Española.

[B22] De la Vega R. (2003). La importancia del entrenamiento de la concentración en el fútbol base: Una perspectiva aplicada. *Cuad. Psicol. Deport.* 3 67–82.

[B23] Desiderio D. W. A. Bortolazzo C. (2020). Impacto de la pandemia por covid-19 en los deportistas. *Rev. Asoc. Méd. Argent.* 133 187–189.

[B24] Di Fronso S. Costa S. Montesano C. Di Gruttola F. Ciofi E. G. Morgilli L. (2020). The effects of COVID-19 pandemic on perceived stress and psychobiosocial states in Italian athletes. *Int. J. Sport Exerc. Psychol.* 1–13. 10.1080/1612197X.2020.1802612

[B25] Epidemiology Working Group for NCIP Epidemic Response, Chinese Center for Disease Control and Prevention. (2020). The epidemiological characteristics of an outbreak of 2019 novel coronavirus diseases (COVID-19) in China. *Zhonghua Liu Xing Bing Xue Za Zhi* 41 145–151. 10.3760/cma.j.issn.0254-6450.2020.02.003 32064853

[B26] Evans A. B. Blackwell J. Dolan P. Fahlén J. Hoekman R. Lenneis V. (2020). Sport in the face of the COVID-19 pandemic: towards an agenda for research in the sociology of sport. *Eur. J. Sport Soc.* 17 85–95. 10.1080/16138171.2020.1765100

[B27] Fuentes I. Balaguer I. Meliá J. L. García-Merita M. L. (1995). *Forma Abreviada del Perfil de los Estados de Ánimo (POMS). Actas del V Congreso Nacional de Psicología de la Actividad Física y el Deporte.* Valencia: Universidad de Valencia 29–39.

[B28] Gandek B. Ware J. E. Aaronson N. K. Apolone G. Bjorner J. B. Brazier J. E. (1998). Crossvalidation of item selection and scoring for the SF-12 health survey in nine countries: results from the IQOLA project. *Int. Qual. Life Assess. J. Clin. Epidemiol.* 51 1171–1178. 10.1016/s0895-4356(98)00109-7 9817135

[B29] González-Sanguino C. Ausín B. Castellanos M. Á Saiz J. López-Gómez A. Ugidos C. (2020). Mental health consequences during the initial stage of the 2020 Coronavirus pandemic (COVID-19) in Spain. *Brain Behav. Immun.* 87 172–176. 10.1016/j.bbi.2020.05.040 32405150 PMC7219372

[B30] Graupensperger S. Benson A. J. Kilmer J. R. Evans M. B. (2020). Social (un) distancing: teammate interactions, athletic identity, and mental health of student-athletes during the COVID-19 pandemic. *J. Adolesc. Health* 67 662–670. 10.1016/j.jadohealth.2020.08.001 32943294 PMC7489994

[B31] Graupensperger S. Lee C. M. Larimer M. E. (2021). Young adults underestimate how well peers adhere to COVID-19 preventive behavioral guidelines. *J. Prim. Prev.* 42 309–318. 33932222 10.1007/s10935-021-00633-4 PMC8088206

[B32] Gross J. Ligges U. (2015). *nortest: Tests for Normality. R package version 1.0-4.* Available online at: https://CRAN.R-project.org/package=nortest (accessed April 25, 2021).

[B33] Gupta S. McCarthy P. J. (2021). Sporting resilience during COVID-19: what is the nature of this adversity and how are competitive elite athletes adapting? *Front. Psychol.* 12:611261. 10.3389/fpsyg.2021.611261 33746833 PMC7966721

[B34] Ivarsson A. Johnson U. Andersen M. B. Tranaeus U. Stenling A. Lindwall M. (2017). Psychosocial factors and sport injuries: meta-analyses for prediction and prevention. *Sports Med.* 47 353–365. 10.1007/s40279-016-0578-x 27406221

[B35] Izzicupo P. Di Baldassarre A. Abelkalns I. Bisenieks U. Sánchez-Pato A. Cánovas Álvarez F. J. (2021). Dual career of athletes during COVID-19 lockdown. *Front. Psychol.* 12:739. 10.3389/fpsyg.2021.657671 33868131 PMC8047065

[B36] Jukic I. Calleja-González J. Cos F. Cuzzolin F. Olmo J. Terrados N. (2020). Strategies and Solutions for Team Sports Athletes in Isolation due to COVID-19. *Sports (Basel, Switzerland)* 8 56. 10.3390/sports8040056 32344657 PMC7240607

[B37] Kalazich Rosales C. Valderrama Erazo P. Flández Valderrama J. Burboa González J. Humeres Terneus D. Urbina Stagno R. (2020). Orientaciones Deporte y COVID-19: Recomendaciones sobre el retorno a la actividad física y deportes de niños niñas y adolescentes. *Rev. Chil. Pediatr.* 91 75–90.

[B38] Kassambara A. (2021). *rstatix: Pipe-Friendly Framework for Basic Statistical Tests. R package version 0.7.0.* Available online at: https://CRAN.R-project.org/package=rstatix (accessed June 7, 2021).

[B39] Lades L. Laffan K. Daly M. Delaney L. (2020). Daily emotional well-being during the COVID-19 pandemic. *Br. J. Health Psychol.* 25 902–911. 10.31234/osf.io/pg6bw 32573074 PMC7361840

[B40] Lima C. K. T. de Medeiros Carvalho P. M. Lima I. D. A. S. de Oliveira Nunes J. V. A. Saraiva J. S. de Souza R. I. (2020). The emotional impact of coronavirus 2019-nCoV (new coronavirus disease). *Psychiatry Res.* 287 112915. 10.1016/j.psychres.2020.112915 PMC7195292 32199182

[B41] López de Subijana C. Barriopedro M. Conde E. (2015). Supporting dual career in Spain: Elite athletes’ barriers to study. *Psychol. Sport Exerc.* 21 57–64. 10.1016/j.psychsport.2015.04.012

[B42] Mantilla S. C. Gómez-Conesa A. (2007). El Cuestionario Internacional de Actividad Física. Un instrumento adecuado en el seguimiento de la actividad física poblacional. *Rev. Iberoam. Fisioter. Kinesiol.* 10 48–52. 10.1016/S1138-6045(07)73665-1

[B43] McGuine T. A. Biese K. M. Petrovska L. Hetzel S. J. Reardon C. Kliethermes S. (2021). Mental health, physical activity, and quality of life of us adolescent athletes during COVID-19–related school closures and sport cancellations: a study of 13 000 athletes. *J. Athl. Train.* 56 11–19. 10.4085/1062-6050-0478.20 33290516 PMC7863599

[B44] McNair D. M. Lorr M. Droppelman L. F. (1992). *Manual for the Profile of Mood States.* San Diego, CA: Educational and Industrial Testing Service.

[B45] Mera A. Y. Tabares-Gonzalez E. Montoya-Gonzalez S. Muñoz-Rodriguez D. I. Vélez F. M. (2020). Recomendaciones prácticas para evitar el desacondicionamiento físico durante el confinamiento por pandemia asociada a COVID-19. *Universidad Salud* 22 166–177. 10.22267/rus.202202.188

[B46] Meza E. I. A. López J. A. H. (2021). Physical activity in university student athletes, prior and in confinement due to pandemic associated with COVID-19. *Retos* 39 572–575.

[B47] Montero F. J. O. Tenza E. O. Cano L. A. Hernández J. G. Vera P. J. (2014). Influencia de la lesión en la vida deportiva y personal del deportista, y propuestas de intervención. *Rev. Psicol. Deport.* 23 465–471.

[B48] Mohr M. Nassis G. P. Brito J. Randers M. B. Castagna C. Parnell D. (2020). Return to elite football after the COVID-19 lockdown. *Manag. Sport Leis.* 1–9.

[B49] Mujika I. Halson S. Burke L. M. Balagué G. Farrow D. (2018). An integrated, multifactorial approach to periodization for optimal performance in individual and team sports. *Int. J. Sports Physiol. Perform.* 13 538–561. 10.1123/ijspp.2018-0093 29848161

[B50] Olmedilla A. Ortega E. Gómez J. M. (2014). Influencia de la lesión deportiva en los cambios del estado de ánimo y de la ansiedad precompetitiva en futbolistas. *Cuad. Psicol. Deport.* 14 55–62.

[B51] Pallarés S. Azócar F. Torregrosa M. Selva C. Ramis Y. (2011). Modelos de trayectoria deportiva en waterpolo y su implicación en la transición hacia una carrera profesional alternativa. (Athletic Career Models in Water Polo and their Involvement in the Transition to an Alternative Career). *Cultura Cienc. Deport.* 6 93–103. 10.12800/ccd.v6i17.36

[B52] Pillay L. van Rensburg D. C. C. J. van Rensburg A. J. Ramagole D. A. Holtzhausen L. Dijkstra H. P. (2020). Nowhere to hide: the significant impact of coronavirus disease 2019 (COVID-19) measures on elite and semi-elite South African athletes. *J. Sci. Med. Sport* 23 670–679. 10.1016/j.jsams.2020.05.016 32448749 PMC7235602

[B53] Pons J. Ramis Y. Alcaraz S. Jordana A. Borrueco M. Torregrossa M. (2020). Where did all the sport go? negative impact of COVID-19 lockdown on life-spheres and mental health of spanish young athletes. *Front. Psychol.* 11:611872. 10.3389/fpsyg.2020.611872 33365006 PMC7750436

[B54] Remor E. Carrobles J. A. (2001). Versión Española de la escala de estrés percibido (PSS-14): Estudio psicométrico en una muestra VIH+. *Ansiedad Estrés* 7 195–201.

[B55] Rowe D. (2020). Subjecting pandemic sport to a sociological procedure. *J. Sociol.* 56 704–713. 10.1177/1440783320941284

[B56] Samuel R. D. Tenenbaum G. Galily Y. (2020). The 2020 coronavirus pandemic as a change-event in sport performers’ careers: conceptual and applied practice considerations. *Front. Psychol.* 11:567966. 10.3389/fpsyg.2020.567966 33071895 PMC7540073

[B57] Sanders G. Stevinson C. (2017). Associations between retirement reasons, chronic pain, athletic identity, and depressive symptoms among former professional footballers. *Eur. J. Sport Sci* 17 1311–1318. 10.1080/17461391.2017.1371795 28911275

[B58] Schinke R. J. Papaioannou A. Maher C. Parham W. D. Larsen C. H. Gordin R. (2020). Sport psychology services to professional athletes: working through COVID-19. *Int. J. Sport Exerc. Psychol.* 18 409. 10.1080/1612197X.2020.1766182

[B59] Stambulova N. B. Schinke R. J. Lavallee D. Wylleman P. (2020). The COVID-19 pandemic and Olympic/Paralympic athletes’ developmental challenges and possibilities in times of a global crisis-transition. *Int. J. Sport Exerc. Psychol.* 2020 1–10. 10.1080/1612197X.2020.1810865

[B60] Stambulova N. B. Wylleman P. (2015). Dual career development and transitions. *Psychol. Sport Exerc.* 21 1–134.

[B61] Taku K. Arai H. (2020). Impact of COVID-19 on athletes and coaches, and their values in Japan: repercussions of postponing the Tokyo 2020 olympic and paralympic games. *J. Loss Trauma* 25 623–630.

[B62] Toresdahl B. G. Asif I. M. (2020). Coronavirus disease 2019 (COVID-19): considerations for the competitive athlete. *Sports Health* 12 221–224. 10.1177/1941738120918876 32250193 PMC7222670

[B63] Tran T. T. Lundgren L. Secomb J. Farley O. R. L. Haff G. G. Nimphius S. (2017). Effect of four weeks detraining on strength, power, and sensorimotor ability of adolescent surfers. *Open Sports Sci. J.* 10 71–80. 10.2174/1875399X01710010071

[B64] Vilagut G. Ferrer M. Rajmil M. Rebollo P. Permanyer-Miralda G. Quintana J. M. (2005). El cuestionario de salud SF-36 español: una década de experiencia y nuevos desarrollos. *Gac. Sanit.* 19 135–150. 15860162 10.1157/13074369

[B65] Vilagut G. Valderas J. M. Ferrer M. Garin O. López-García E. Alonso J. (2008). Interpretación de los cuestionarios de salud SF-36 y SF-12 en España: componentes físico y mental. *Med. Clín.* 130 726–735. 10.1157/13121076 18570798

[B66] Wadey R. Evans L. Hanton S. Neil R. (2013). Effect of dispositional optimism before and after injury. *Med. Sci. Sports Exerc.* 45 387–394. 10.1249/MSS.0b013e31826ea8e3 22903140

[B67] Ware J. E. Kosinski M. Keller S. D. (1996). A 12-Item Short-Form Health Survey: construction of scales and preliminary tests of reliability and validity. *Med. Care* 34 220–233. 10.1097/00005650-199603000-00003 8628042

[B68] Ware J. E. Sherbourne C. D. (1992). The MOS 36-item short-form health survey (SF-36) (I). Conceptual framework and item selection. *Med. Care* 30 473–483. 10.1097/00005650-199206000-00002 1593914

[B69] Wei T. Simko V. (2017). *R package “corrplot”: Visualization of a Correlation Matrix (Version 0.84).*

[B70] Woodford L. Bussey L. (2021). Exploring the perceived impact of the COVID-19 pandemic social distancing measures on athlete wellbeing: a qualitative study utilising photo-elicitation. *Front. Psychol.* 12:2727. 10.3389/fpsyg.2021.624023 PMC8311490 34322049

[B71] World Health Organization (2020a). *Coronavirus Disease 2019 (COVID-19): Situation Report- 51. 21 March 2020.* Geneva: World Health Organization.

[B72] World Health Organization (2020b). *Mental Health and Psychosocial Considerations During the COVID-19 Outbreak (No. WHO/2019-nCoV/MentalHealth/2020.1).* Geneva: WHO.

[B73] Xiang Y. T. Yang Y. Li W. Zhang L. Zhang Q. Cheung T. (2020). Timely mental health care for the 2019 novel coronavirus outbreak is urgently needed. *Lancet Psychiatry* 7 228–229. 10.1016/S2215-0366(20)30046-8 32032543 PMC7128153

