# Supplementary material for: Effects of the COVID-19 Health Crisis on Sports Practice, Life Quality, and Emotional Status in Spanish High-Performance Athletes
Source: Front Psychol. 2021 Sep 27;12:736499. doi: 10.3389/fpsyg.2021.736499 (PMC8503513; doi:10.3389/fpsyg.2021.736499)
Supplement: Supplementary file 1 [file Data_Sheet_1.PDF]

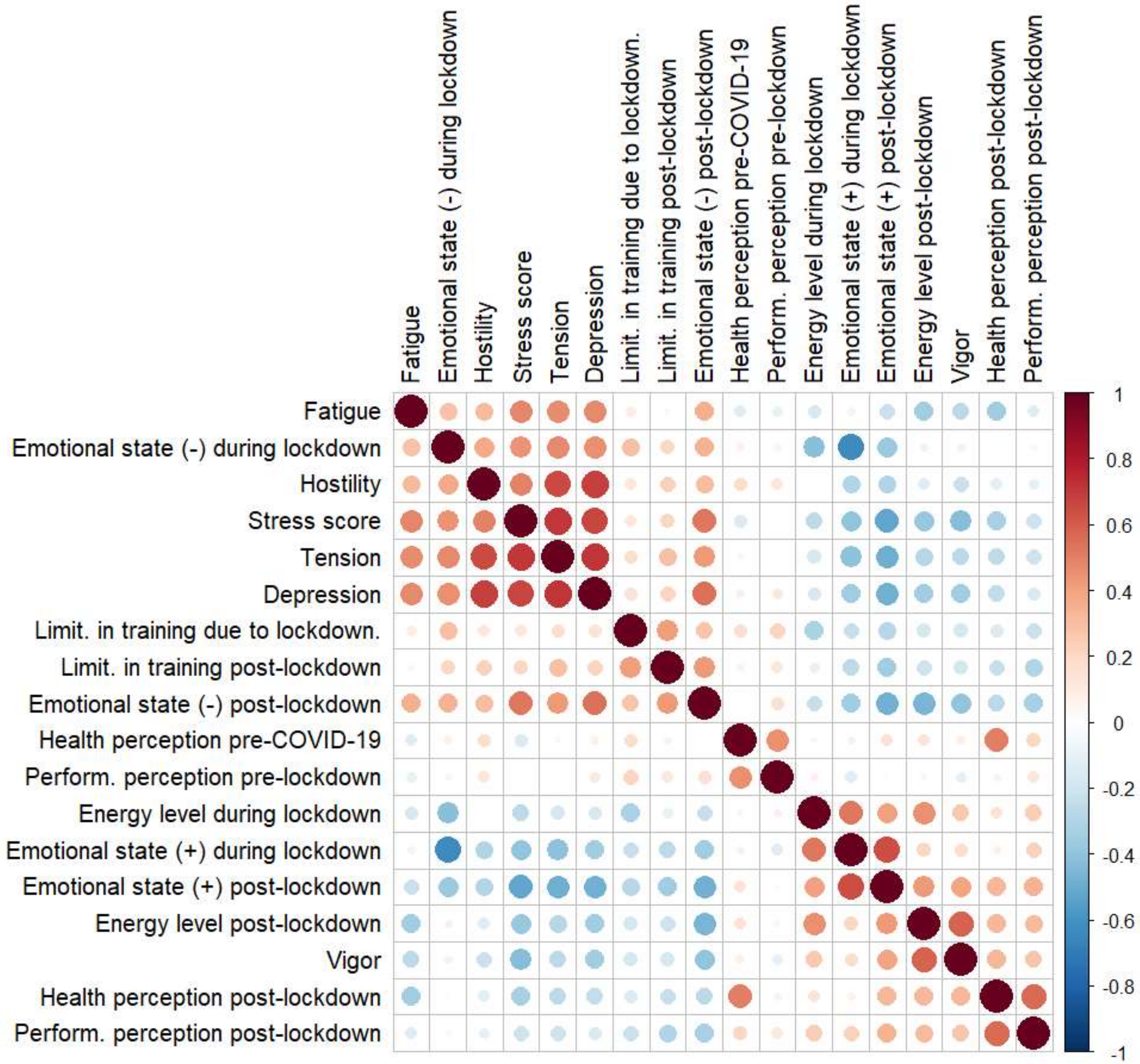


**Supplemental Figure 1.** Correlogram depicting the correlations among stress, POMS, emotional state and energy level, health and performance perception, and limitation in training variables. Positive correlations are displayed in red and negative correlations in blue. Size of the circle and colour intensity are proportional to the correlation coefficients.
